# Extracellular Proximity Labeling Reveals an Expanded Interactome for the Matrisome Protein TIMP2

**DOI:** 10.21203/rs.3.rs-3857263/v1

**Published:** 2024-01-15

**Authors:** David Peeney, Sadeechya Gurung, Josh A. Rich, Sasha Coates-Park, Yueqin Liu, Jack Toor, Jane Jones, Christopher T. Richie, Lisa M. Jenkins, William G. Stetler-Stevenson

**Affiliations:** 1 Laboratory of Pathology, Center for Cancer Research, National Cancer Institute, Bethesda, MD, USA.; 2 Center for Cancer Research Protein Expression Laboratory, National Cancer Institute, Frederick, MD, USA.; 3 Genetic Engineering and Viral Vector Core, Office of the Scientific Director, National Institute on Drug Abuse, Baltimore, MD, USA.; 4 Laboratory of Cell Biology, Center for Cancer Research, National Cancer Institute, Bethesda, MD, USA

## Abstract

Classical methods of investigating protein-protein interactions (PPIs) are generally performed in non-living systems, yet in recent years new technologies utilizing proximity labeling (PL) have given researchers the tools to explore proximal PPIs in living systems. PL has distinct advantages over traditional protein interactome studies, such as the ability to identify weak and transient interactions in vitro and in vivo. Most PL studies are performed on targets within the cell or on the cell membrane. We have adapted the original PL method to investigate PPIs within the extracellular compartment, using both BioID2 and TurboID, that we term extracellular PL (ePL). To demonstrate the utility of this modified technique, we investigate the interactome of the widely expressed matrisome protein tissue inhibitor of metalloproteinases 2 (TIMP2). Tissue inhibitors of metalloproteinases (TIMPs) are a family of multi-functional proteins that were initially defined by their ability to inhibit the enzymatic activity of metalloproteinases (MPs), the major mediators of extracellular matrix (ECM) breakdown and turnover. TIMP2 exhibits a broad expression profile and is often abundant in both normal and diseased tissues. Understanding the functional transformation of matrisome regulators, like TIMP2, during the evolution of tissue microenvironments associated with disease progression is essential for the development of ECM-targeted therapeutics. Using carboxyl- and amino-terminal fusion proteins of TIMP2 with BioID2 and TurboID, we describe the TIMP2 proximal interactome. We also illustrate how the TIMP2 interactome changes in the presence of different stimuli, in different cell types, in unique culture conditions (2D vs 3D), and with different reaction kinetics (BioID2 vs. TurboID); demonstrating the power of this technique versus classical PPI methods. We propose that the screening of matrisome targets in disease models using ePL will reveal new therapeutic targets for further comprehensive studies.

## Introduction

The extracellular matrix (ECM) is a complex framework that supports all cellular and tissue processes and is made up of over 1000 proteins referred to as the matrisome. It is well established through the principles of dynamic reciprocity, which define the bidirectional interaction between cells and the extracellular space, that the ECM directly modulates cell behavior [1]. These principles are mediated, in large part, through protein-protein interactions linking the extracellular and intracellular compartments. Virtually all mammalian disease states have roots entrenched within the ECM, manifesting as abnormal abundance or dysfunctional activity of matrisomal proteins [2]. Despite widespread appreciation of the significant alterations in the disease-associated matrisome, therapeutic targeting of the ECM has shown limited success across the spectrum of human diseases.

Tissue inhibitors of metalloproteinases (TIMPs) are a family of endogenous matrisome proteins that were initially defined by their ability to inhibit the enzymatic activity of metalloproteinases (MPs), the major mediators of ECM breakdown and turnover [3]. In turn, this function of TIMPs makes them important regulators of ECM structure. Since their original discovery, various additional MP-independent functions have been attributed to TIMP family members leading to their designation as multifunctional proteins with discrete functional domains [4]. This can be exemplified by TIMP2, the most abundant member of the family that possesses a range of pro- and anti-mitogenic functions that are independent of its MP inhibitory activity [5]. Due to their multifunctional capabilities, the therapeutic potential of TIMP proteins across various conditions has been proposed in recent years. We and others have illustrated how TIMP2 exhibits anti-tumor capabilities [6–9], and others have described how TIMP2 may protect against age-related neuronal dysfunction [10–12]. Biochemically, TIMP2 is a promiscuous protein, binding to and inhibiting all 23 members of the matrix metalloproteinase (MMP) family as well as ADAM12 [13] and ADAMTS8 [14] and targeting several membrane proteins [5]. Despite the therapeutically promising capabilities of TIMP2, many of the detailed mechanisms reinforcing these functions are missing. Exploration of the TIMP2 interactome using affinity purification methods has yielded incomplete results, the limitations of which are well described [15].

A powerful method for identifying PPIs is proximity labeling (PL), which utilizes target fusions with promiscuous biotin ligases such as BioID2/TurboID [16]. These fusions preferentially biotinylate interacting partners that can be identified using basic enrichment steps followed by mass spectrometry. The majority of studies utilizing biotin ligases target intracellular proteins.

Using the broadly expressed matrisome protein TIMP2, we illustrate that conditions can be optimized for proximity labeling reactions in the extracellular milieu. Using TIMP2 fusions with BioID2 and TurboID, we identify novel proximal interactors for TIMP2 across two different cell types and in a range of culture conditions, providing new context regarding TIMP2 functions in health and disease. Furthermore, our workflow serves as a template for proximal interactome studies of other matrisome targets, representing an important technical resource for extracellular matrix biologists.

## Results

### TIMP2 as an ideal candidate for extracellular proximity labeling (ePL)

**Figure 1.**
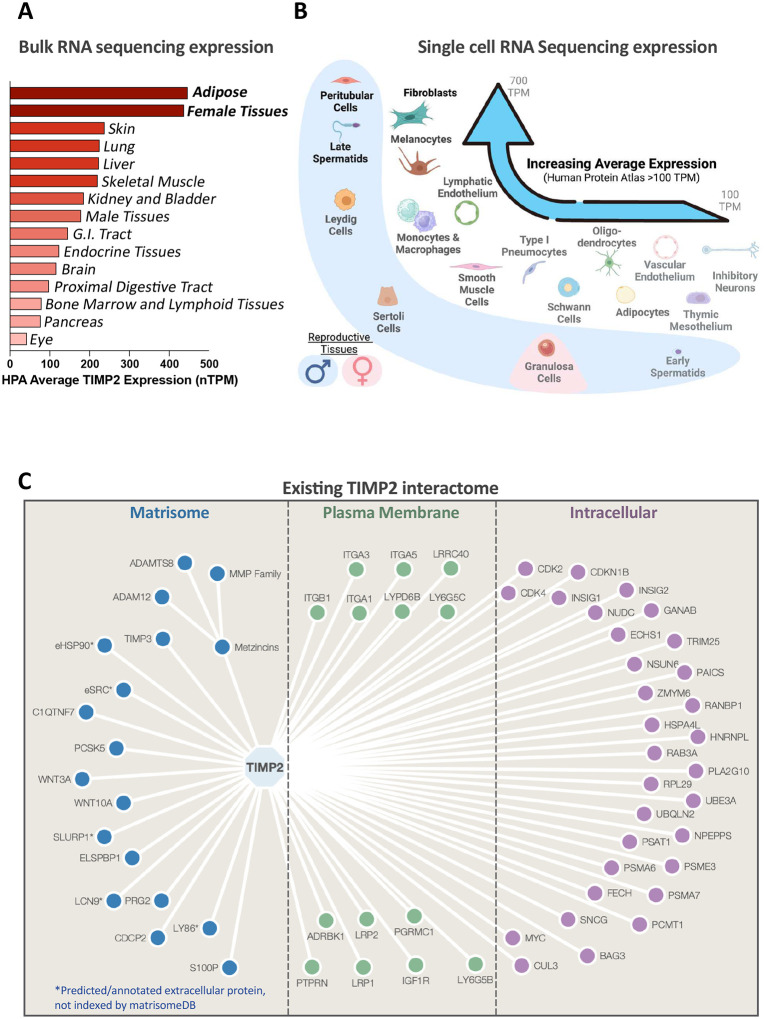
Tissue Inhibitor of Metalloproteinase 2 as an ideal candidate for extracellular proximity labeling. (A) Average TIMP2 transcript expression (normalized transcripts per million; nTPM) from bulk RNA sequencing data analyzed by the Human Protein Atlas across a range of human tissues. (B) Graphic interpretation of single cell RNA sequencing data processed by the Human Protein Atlas. Cells are ordered by average expression, with a minimum average expression threshold of 100 TPM. Created with BioRender.com. (C) Diagram of known TIMP2 interactors harvested from IntAct, BioGRID, STRING, BioPlex, and through a manual literature search.

TIMP2 is a 22kDa matrisome protein that has well described structural and functional characteristics [5]. Query of human TIMP2 transcript expression through the Human Protein Atlas reveals a broad RNA expression profile, with a distinct enrichment in adipose and female tissues (Figure 1A) [17]. Furthermore, analysis of single cell RNA sequencing data, also of the Human Protein Atlas, illustrates that TIMP2 is widely expressed across various cell types, with a particular enrichment in cells of mesodermal lineages such as fibroblasts (Figure 1B) [18]. Of note is the strong expression for TIMP2 that is observed across reproductive tissues, particularly of male origin; contrary to Figure 1A. We harvested TIMP2 interactome data from several databases (IntAct; www.ebi.ac.uk/intact, BioGRID; thebiogrid.org, STRING; string-db.org, BioPlex; bioplex.hms.harvard.edu) [19–22] and compiled the reported interactors based on their specified tissue location (Figure 1C, Table S1). Tissue locations were determined empirically using the MatrisomeDB, GeneCards, and literature searches [23–25]. Some of the reported interactors that we designated as matrisome proteins are not indexed by the MatrisomeDB, since we included secreted proteins in this designation for simplicity. These include SLURP1, LCN9, LY86, or proteins that are classically intracellular proteins but demonstrate extracellular forms that exhibit TIMP2 binding (HSP90, SRC) [26, 27].

**Figure 2.**
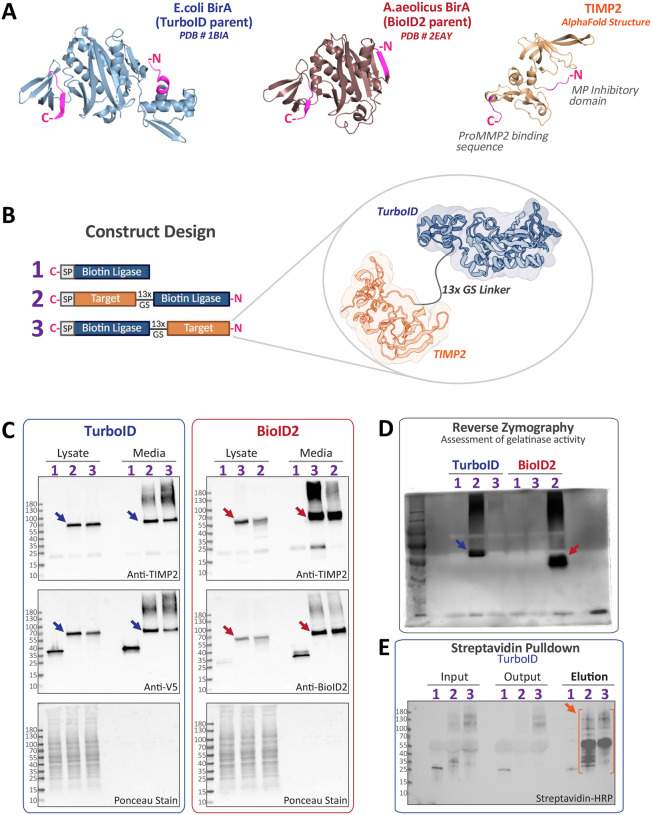
Expression, secretion, and function of biotin ligase fusion proteins. (A) Structure of E.coli BirA (parental TurboID protein), A.aeolicus BirA (parental BioID2 protein), and the AlphaFold predicted structure of TIMP2. (B) Basic construct design and proposed structure of one fusion protein. (C) Immunoblots assessing the expression and secretion of fusion proteins in HT1080 cells. (D) Reverse zymography (10% acrylamide) of conditioned media from fusion protein expressing cells revealing retention of MMP-inhibitory activity in a single fusion orientation. (E) Streptavidin pulldown and blotting of SDS-PAGE nitrocellulose blots with streptavidin-HRP reveals unique patterns of biotinylated proteins in fusion protein expressing samples.

BirA is a bi-functional protein that acts as a biotin ligase and transcriptional repressor, which is well conserved in single-celled organisms [28]. The first engineered version, BirA*, was based on BirA from E. coli that possessed an individual point mutation (R118G) that enabled promiscuous biotinylation [29, 30]. The characteristics of BirA* were ultimately exploited for proximity labeling studies with the described technique named proximity-dependent biotin identification (BioID) [31]. These original studies spawned the search for more effective alternatives, including TurboID that was developed based on directed evolution of the original BirA* [32]. BioID2 was developed using BirA from A. aeolicus, which is substantially smaller than E. coli BirA at 27 kDa (versus 35 kDa) [33]. This reduced size is largely the consequence of a truncated N-terminal DNA-binding domain that does not significantly impact biotin ligase activity. The crystal structure of the parental forms of TurboID (PDB 1BIA, [34]) and BioID2 (PDB 2EAY), in comparison to that of TIMP2 (AlphaFold prediction, [35]), suggested to us that generating contrasting orientations of TIMP2:biotin ligase fusion proteins would reveal a more complete proximal interactome (Figure 2A). Constructs encoding fusion proteins of both orientations, separated by a flexible 13x GGGGS-linker (GS-linker) sequence to increase reactive radius, were produced containing an N-terminal appended secretory signal sequence to promote secretion into the extracellular compartment (Figure 2B). Retroviral-mediated fusion protein expression was analyzed by immunoblotting, confirming the successful secretion and comparable production of fusion proteins (Figure 2C). The fusions retained important functional characteristics, including the ability to inhibit MMP2 activity in the fusion orientation with a free TIMP2 N-terminus (Figure 2D). Streptavidin horseradish peroxidase staining of pulldown samples (input, output, and elution) reveals a unique pattern of biotinylated proteins detected in TIMP2-TurboID fusion protein samples, indicative of a functional proximity labeling protocol (Figure 2E).

### ePL reveals the TIMP2 proximal interactome in HT1080 cells

**Figure 3.**
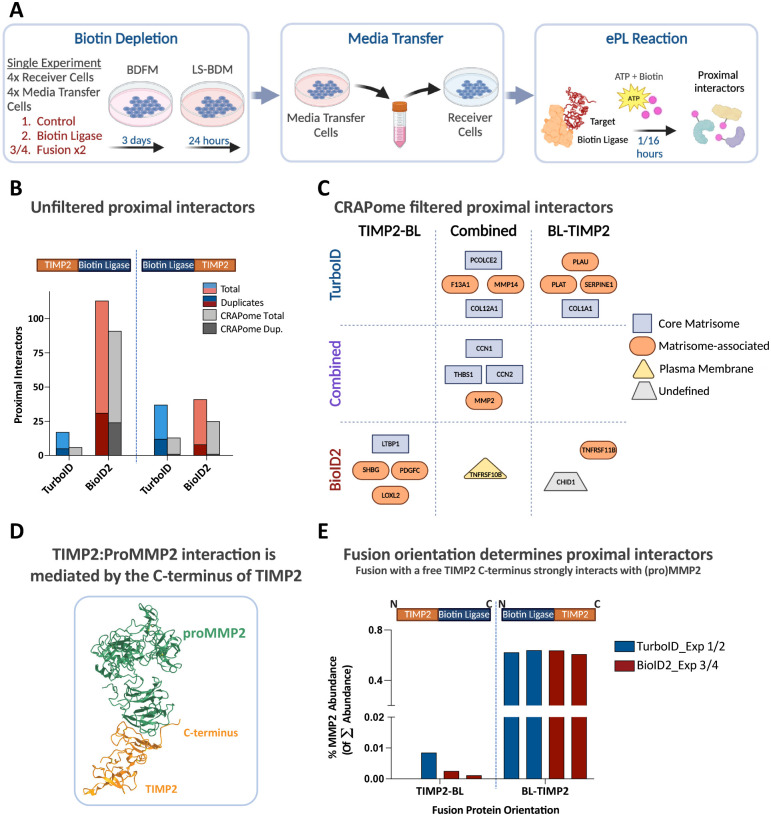
Extracellular proximity (ePL) labeling to identify the proximal interactome of TIMP2 in HT1080 cells. (A) Schematic describing the basic workflow of ePL reactions in HT1080 cells. (B) Data acquired through LC-MS/MS was scored and proximal interactors identified using a defined scoring system. (C) Proximal interactors were uploaded into the Contaminant Repository for Affinity Purification-mass spectrometry (CRAPome) and persistent contaminants were removed based on a defined threshold (identified in >99 control experiments). Duplicate experiment identified proximal interactors are illustrated in the quadrant based on the biotin ligase utilized and the fusion protein orientation. (D) Crystal structure of the TIMP2:proMMP2 complex (Protein Data Bank #1GXD) reveals a strong C-terminal interaction that is (E) significantly perturbed in the presence of a C-terminal fused biotin ligase. (A+C) Created with BioRender.com.

HT1080 cells are an epithelial-like fibrosarcoma cell line that has been extensively utilized by researchers studying the biology of TIMPs and MPs [36–38]. Considering the strong correlation between TIMP2 expression and mesenchymal cells [39] (Figure 1B), they represent an ideal cell line for preliminary ePL studies. A total of 4 experiments were performed, representing duplicates from TurboID and BioID2 variations, fully summarized in Table S2. For TurboID experiments we utilized a 1-hour labeling protocol, whereas for BioID2 labeling was performed over 16 hours (Figure 3A). Surprisingly, despite its higher labeling activity we found that TurboID produced a more limited proximal interactome with a smaller contribution of contaminants defined through contaminant repository for affinity purification (CRAPome) analysis (Figure 3B) [40]. A core of 4 proteins (CCN1, CCN2, THBS1, MMP2) were identified as proximal interactors in each experiment (Figure 3C). Three of these are potentially novel interactors for TIMP2, with the TIMP2-MMP2 interaction being well characterized [41]. Across all experiments, at least 19 candidate interactors were identified through duplicate identification, some displaying orientation specificity and others biotin ligase specificity. The orientation specificity of the proximal interactome can be appreciated through the TIMP2:proMMP2 interaction that requires a free C-terminal tail of TIMP2, the crystal structure of which is defined in Figure 3D [42]. Initial observations suggest both orientations display proximal interactions with MMP2. This is to be expected, considering that TIMP2 can inhibit active MMP2 via its opposing (N-)terminus. However, the detected MMP2 peptides from the TIMP2-BL data exhibit poor sequence coverage that do not correspond to peptides of the prodomain of MMP2 (0, 6, 7, and 5% sequence coverage versus ≥44% for BL-TIMP2 samples) (Table S2, tab 24). Quantification of the total abundance between the opposing orientations clearly demonstrate that the TIMP2:MMP2 proximal interaction is favored by a free C-terminus of TIMP2 (Figure 3E). Furthermore, the TIMP2:MMP14 interaction is mediated exclusively through the N-terminus of TIMP2 and this is solely detected by the TIMP2-TurboID fusion. Interestingly, the same interaction is not detected by the TIMP2-BioID2 fusion, revealing an important consideration with regards to reaction kinetics and the identification of proximal interactors. The TIMP2:MMP14 interaction has been reported to result in rapid internalization and, to an undefined extent, degradation of the complex [43, 44], possibly explaining the inability of the slower labeling BioID2 fusions to successfully biotinylate MMP14. Unsurprisingly, most of the candidate proximal interactors are matrisome proteins including F13A1 (Coagulation Factor XIII A Chain). RNA sequencing data confirms that HT1080 cells do not express F13A1 transcripts despite its significance in TurboID experiments with both orientations (data available through GEO GSE252575). Assessment of the peptides detected from human F13A1 and alignment with analogous regions in bovine F13A1 reveals the peptides correspond with regions of identical m/z ratio (with single residue differences represented by a switch between leucine and isoleucine) (Figure S2, tab 25). The observation that bovine serum contaminants are identified as proximal interactors presents as both a technical limitation and a source of intrigue, especially considering that TIMP2 is detectable in the human plasma at a concentration of approximately 110ng/mL according to the Human Protein Atlas [45]. Analysis of the raw spectral files searched against the Bos taurus protein database reveals four further proximal interactors for the TurboID:TIMP2 orientation; SERPINA5, CFH, C3, and IGFBP3 (Table S2, tab 26).

### Proximal interactomes are dynamic

**Figure 4.**
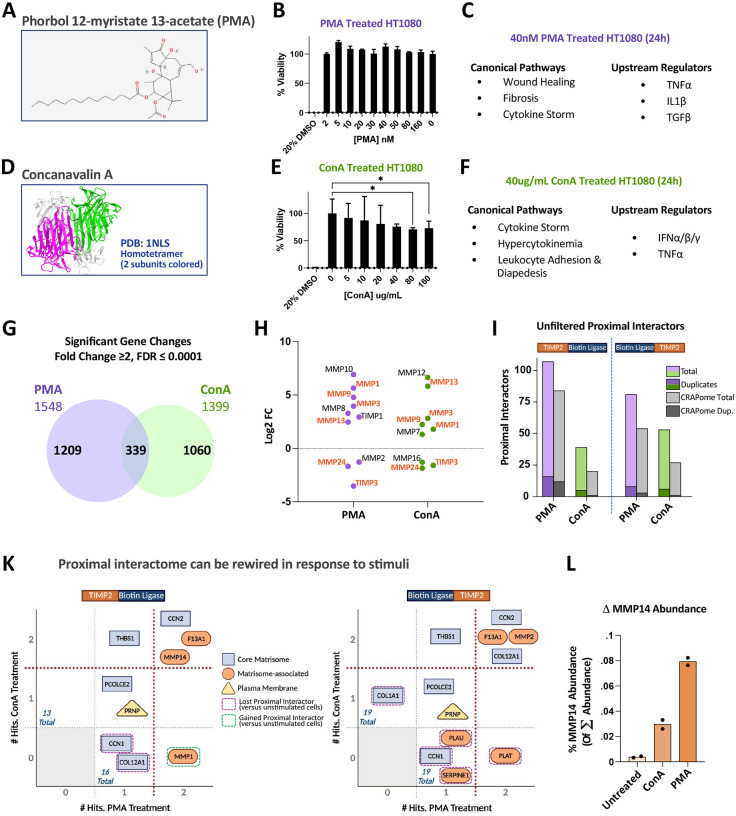
Cellular treatments can reveal proximal interactome dynamics. (A) PubChem structure of phorbol 12-myristate 13-acetate (PMA). (B) PMA displays limited cytotoxicity in HT1080 cells over 24 hours via MTT assay. (C) Ingenuity Pathway Analysis canonical pathways and upstream regulators of transcriptome data from HT1080 cells treated with 40nM PMA for 24 hours. (D) Structure of concanavalin A (ConA). (E) ConA shows a dose-dependent increase in cytotoxicity in HT1080 cells that begins at a concentration above 40ug/mL via MTT assay. (F) Ingenuity Pathway Analysis canonical pathways and upstream regulators of transcriptome data from HT1080 cells treated with 40ug/mL ConA for 24 hours. (G) Venn diagram comparing the transcriptome changes in PMA versus ConA treated HT1080 cells. (H) Example metalloproteinase and TIMP transcript changes in PMA versus ConA treated HT1080 cells. (I) Quantified ePL data using TurboID fusions with TIMP2 acquired through LC-MS/MS that was processed, scored, and proximal interactors identified using a defined system. (K) Tables illustrating the identified TIMP2 proximal interactors (TurboID) in HT1080 cells treated with PMA or ConA, created with BioRender.com. (L) Comparison of the normalized abundance of MMP14 across HT1080 TurboID ePL assays that were untreated, or treated with ConA or PMA.

An important consideration when probing proximal interactomes is that protein-protein interactions are dynamic phenomena, the occurrence of which may have few or many dependencies. These dependencies may include the expression of co-factors or induction of unique post-translational modifications. Phorbol 12-myristate 13-acetate (PMA, Figure 4A) is a plant-derived (*Croton tiglium*) phorbol ester that mimics diacylglycerol, leading to an acute constitutive activation of protein kinase C [46]. The ensuing pathway dysregulation supports an acute inflammatory response that is a hallmark of PMA’s tumor-promoting capabilities [47]. Despite its acute inflammatory effects, PMA is not particularly cytotoxic to HT1080 cells over 24 hours (Figure 4B). Concanavalin A (ConA) is a carbohydrate binding protein (lectin) isolated from the Jack bean (*Canavalia ensiformis*) that binds to α-mannopyranosyl and α-glucopyranosyl residues of glycoproteins and glycolipids. ConA is a homotetrametric protein at neutral pH, forming homodimers at acidic pHs [48–50] (Figure 4D). At concentrations of 40ug/mL and below, ConA displays limited effects on HT1080 cell viability/growth (Figure 4E). Interactions between lectins and glycoproteins at the plasma membrane are involved in cellular-ECM interactions that promote ECM remodeling through matrisome regulators such as MMP14 (also known as MT1-MMP), thus ConA is commonly used to stimulate the expression and activity of MMP14 [51]. In the same manner, the inflammatory response induced by PMA also induces the activity of MMP14 [52], which can be supported by gelatin zymography analysis of HT1080 cells treated with ConA and PMA revealing enhanced MMP2 activation (Figure S1), an occurrence that strongly correlates with MMP14 activity [53]. The interaction between MMP14 and TIMP2 is well-studied and has been shown to mediate the activation of MMP2 via formation of a trimolecular complex [53, 54]. Furthermore, this interaction can promote signaling cascades through activation of the MAP kinase and AKT kinase pathways [55, 56]. RNA sequencing corroborates the well-established pro-inflammatory effects of PMA and ConA, which interestingly share common pathways and key upstream regulators despite inducing unique transcriptomic signatures (Figure 4C, 4F, & 4G). In the realm of metalloproteinase activity and regulation, there are several notable transcript level changes in MMPs and TIMPs that are indicative of a change in the balance between MMPs and TIMPs that may shift the TIMP2 proximal interactome (Figure 4H). Using TurboID as the biotin ligase, a series of ePL experiments were performed to determine the proximal interactome of TIMP2 in HT1080 cells treated with 40nM PMA or 40ug/mL ConA, summarized in Table S3. Analysis of the unfiltered proximal interactors reveals a distinct elevation in PMA-induced TIMP2 proximal interactors that are common contaminants as listed by the CRAPome database (Figure 4I). The contribution of CRAPome contaminants in ePL experiments treated with ConA were less pronounced. Examination of the final filtered proximal interactor candidates reveals that, although several of the original interacting partners remain, others are lost or gained (summarized in Figure 4K). Of note is the identification of MMP1 as an interactor for the TIMP2-TurboID orientation in the PMA treatment only, despite MMP1 being upregulated in both cell treatments (Figure 4H & 4K). Furthermore, although MMP14 is detected in all TurboID experiments as an interactor for TIMP2-TurboID, assessment of the normalized abundance reveals that the detection of MMP14 as a proximal interactor increases following treatment with ConA and, to a larger extent, PMA (Figure 4L). This observation is consistent with zymography that illustrates PMA is a more effective activator of MMP2 than ConA in HT1080 cells, an established functional consequence of MMP14 activity that was described earlier (Figure S1).

**Figure 5.**
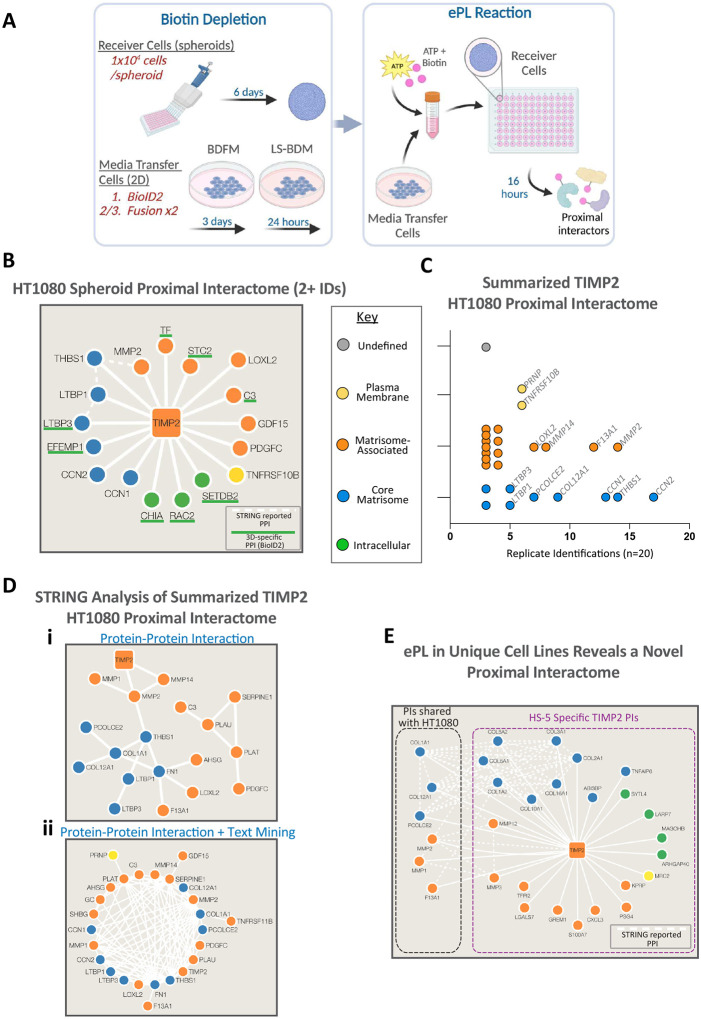
ePL can be performed across multiple live in vitro systems. (A) Basic workflow of HT1080 ePL experiments performed in 3D as spheroids, created with BioRender.com. (B) Summary of TIMP2 proximal interactors (BioID2) identified in 3D spheroid cultures, compared with previous TIMP2-BioID2 ePL experiments. (C) Overall summary of the TIMP2 proximal interactome in HT1080 cells, a total of 5 experiments each performed in duplicate with both fusion orientations using both TurboID and BioID2. (D) STRING analysis of the HT1080 proximal interactome for TIMP2 reveals potential interactome neighborhoods using previously reported direct protein-protein interactions, and by incorporating potential interactions through STRING’s text mining feature. (E) TIMP2 ePL experiments with TurboID in HS-5 bone marrow stromal cells reveals shared and novel interactors.

Cell culture models propagated in 2-dimensions are widely used because they are simple and economical. However, this simplicity comes with many limitations, the most important being a loss in physiological relevance. Cell cultures grown in 3-dimensions help to address this limitation by supporting extensive cell-cell and cell-ECM interactions and facilitating cell and tissue polarity [57]. Spheroid models are the simplest of the 3D culture methods, with scaffold-free cell clumping induced through gravity (hanging drop method) or low-binding plastic. This method relies on cells to secrete their own ECM to support spheroid structure, which can take up to 7 days, although some cell lines are not amenable to this method. HT1080 cells produce robust spheroids that form within 6 days of culture, so we used this system as proof-of-principle that ePL is effective in 3D, summarized in Figure 5A. We utilized the BioID2 fusion proteins for these experiments under the assumption that the fusion proteins will require over 1 hour to penetrate the spheroid fully. Furthermore, the extended processing time when harvesting 3D cultures are ill-suited to the rapid labeling that occurs in TurboID experiments. Results from the single duplicate experiment (described as n=4 when considering both fusion protein orientations) reveals there is fair agreement within the predominant proximal interactors between 2D and 3D experiments. A total of 17 proximal interactors were identified in 3D experiments, with 4 proteins being identified in 3+ samples (MMP2, CCN1, LTBP1, LTBP3). Of the 17 proximal interactors, 8 were identified specifically in 3D ePL experiments (LTBP3, CHIA, RAC2, C3, EFEMP1, SETDB2, TF, STC1; Figure 5B, summarized in Table S4).

Summarizing all the experiments from HT1080 cells, we can visualize the predominant proximal interactors for TIMP2 (Figure 5C, Table S5). Unsurprisingly, members of the matrisome represent the major interacting partners for TIMP2. Of the proximal interactors with a total of 5 hits or more, only 2 are known interactors (MMP2 and MMP14), both of which display fusion orientation predilection due to their well-characterized interaction with the C-terminus (latent MMP2) and N-terminus (MMP14, active MMP2) of TIMP2. Analysis of the known PPIs between members of the HT1080 TIMP2 proximal interactome reveals a network of previous reported interactions that may depict local protein complexes or neighborhoods in cultures of HT1080 cells (Figure 5Di). Elaborating on this analysis, the network can be expanded to include a text mining feature that indicates an interaction between proteins that are mentioned together within published scientific abstracts (Figure 5Dii).

It is recognized that protein interactions which occur in core complexes, such as those involved in essential functions, tend to be retained between cell types [22]. In contrast, broader protein interactomes are dynamic and can be rewired between cell types in a manner that ultimately dictates the unique phenotypes of each cell type [22]. In support of the latter point, we show that the proximal interactome for TIMP2 is rewired in HS-5 bone marrow stromal cells (Figure 5E). A total of six proximal interactors are shared with the compiled HT1080 interactome and, like in HT1080, the matrisome makes up the bulk of TIMP2 interactors in HS-5 cells (results summarized in Table S6). Since some of the protein candidates represent bovine serum proteins and TIMP2 is a well-recognized component of human serum, we performed a duplicate TurboID proximity labeling experiment utilizing recombinant fusion proteins and human serum. These studies revealed that TIMP2 displays a unique serum proximal interactome that, from one donor, includes F13A1 as a proximal interactor (Table S7). Other noteworthy candidates identified by these experiments include Secreted Frizzled Related Protein 1 (SFRP1), the proteinase inhibitor Pregnancy-Zone Protein (PZP), Calpain 2 (CAPN2), and Cathepsin G (CTSG).

**Figure 6.**
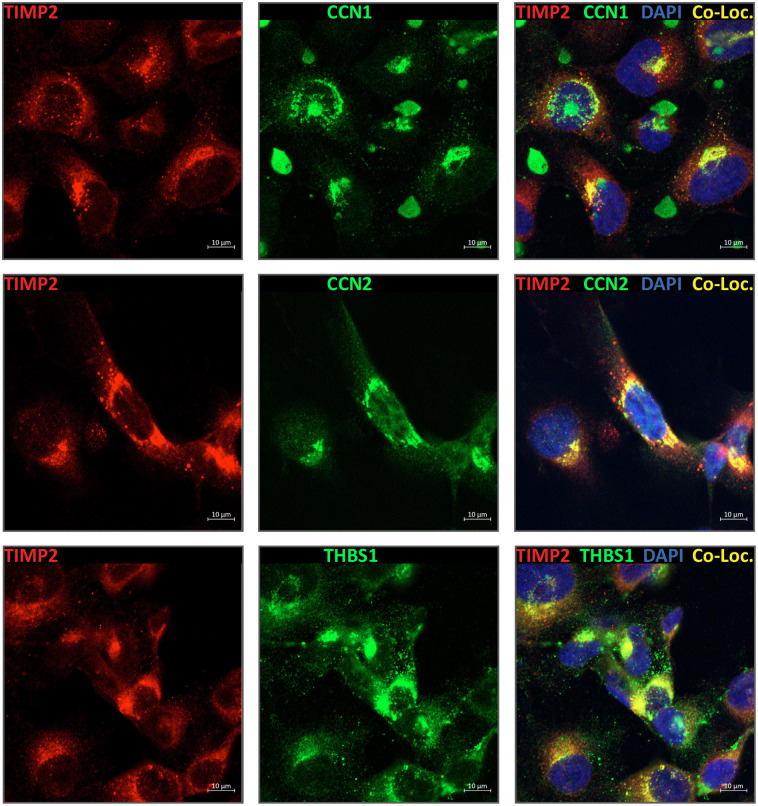
Probing ePL candidate interactors through co-localization studies. HT1080 cells were seeded into glass chamber slides, treated with 0.1ug/mL TIMP2 for 1h, fixed and stained for TIMP2 and CCN1/CCN2/THBS1. Co-localization was assessed through confocal microscopy.

CCN1 (CYR61), CCN2 (CTGF), and THBS1 (TSP1) represent the 3 most common novel TIMP2 proximal interactors identified in our study. Co-immunoprecipitation could not successfully identify either of these targets as direct TIMP2 interactors, an observation that may be influenced by the pitfalls of the technique that requires robust interactions which survive all stages of the protocol. To substantiate our ePL findings, we performed immunofluorescence in HT1080 cells for TIMP2 versus CCN1/CCN2/THBS1, illustrating a distinct co-localization between each target and TIMP2 within the perinuclear region of cells (Figure 6, Figure S2). Indeed, each of these targets have previously been identified in the perinuclear regions of cells, indicating that these targets may share secretory or endocytic pathways [58–63].

## Discussion

Despite great advances in identifying and categorizing the matrisome, a considerable number of its constituents lack thorough functional characterization. These challenges are rooted in the biochemical features of matrisome proteins, in particular members of the core matrisome, that are generally large, insoluble, and structurally linked proteins. Furthermore, members of the matrisome are predisposed to unique post-translational modifications that are uncommon in the broader proteome [64]. The diversity in proteoform contributes to the overwhelming complexity of the matrisome, despite it comprising <10% of the total proteome [23]. Complimentary to the characterization of the matrisome in models of health and disease, knowledge of protein-protein interactions within the extracellular compartment provides vital context with regards to the biological functions of individual components. Due to the complex biochemical properties of matrisome proteins, in particular structural proteins, interrogation of extracellular PPIs is best implemented in living systems [64]. To this end, proximity labeling-based methods are powerful techniques that offer great promise. Historically, these have been used to assess intracellular or plasma membrane proximal interactions, using methods that rely on BirA (BioID), APEX, or biotinylation by antibody recognition (BAR) [16, 65, 66]. The latter technique relies on the generation of truly effective antibodies, representing an additional level of technical complexity. Indeed, the overall performance and consistency of many established antibodies is questioned in a recent study [67]. Using TIMP2 as a model, we provide evidence of the power of ePL when interrogating the proximal interactome of matrisome proteins. TIMP2 is an intriguing target for proximal interactome studies due to its well-defined functions with regards to metalloproteinase interactions, in addition to a slew of mechanistically undefined functions, reviewed extensively elsewhere [5]. We identified a core of highly reproducible proximal interactors that were detected in ≥50% of samples from HT1080 cells: CCN1, CCN2, MMP2, THBS1, and F13A1. All these proximal interactors are members of the matrisome (3 core matrisome, 2 matrisome-associated), with only 1 (MMP2) being a known interactor. To substantiate some of these findings, we performed a series of immunofluorescence experiments to show that TIMP2 co-localizes with the three most identified novel proximal interactors (CCN1/CCN2/THBS1). In each case, TIMP2 co-localized with these targets in the intracellular compartment, specifically in perinuclear localizations. Components of the matrisome are frequently observed to localize in perinuclear regions in immunostaining protocols, possibly linked to migration, invasion, and, specifically, the formation of podosomes and invadopodia—intracellular sites for ECM attachment and degradation [68–70]. Whether the perinuclear co-localization of these targets is a consequence of migratory phenotypes in cells is a question of particular interest.

That ePL proved an effective method for assessing the TIMP2 interactome, despite competing with high levels of endogenous TIMP2, is a testament to its power. For TIMP2, the proximal interactome is not extensively rewired across treatments (untreated, PMA, and ConA) and culture conditions (2D and 3D culture conditions). However, across cell types the proximal interactome is largely unique, despite the cell types sharing a mesodermal lineage. HT1080 is an epithelial-like fibrosarcoma cell line, whereas HS-5 is a genetically transformed bone marrow stromal cell line [36, 71]. The stark differences between the TIMP2 proximal interactome in these cells serves as a testament to the idea of disease-specific PPIs that may convey functional knowledge and/or clinical diagnostic power. This illustrates the importance of choosing the most relevant models when probing proximal interactomes, a feat that will be aided through query of available transcriptomic and proteomic datasets.

**Figure 7.**
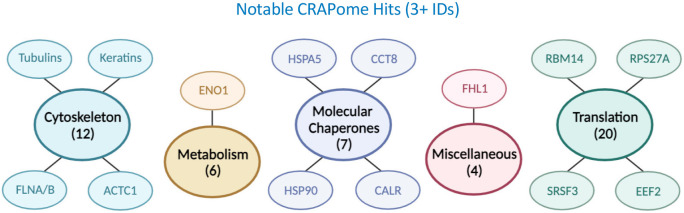
CRAPome filtering can cause data loss through false-negative reporting.

Routinely, the proteins detected in highest abundance are the carboxylases, such as PCCA, PC, MCCC1, ACACA. These enzymes are biotin-dependent, containing a covalently bound biotin through amide linkage between a lysine and the carboxylate moiety of biotin [72]. Another persistent background artefact in our analyses was the incidence of Heat Shock Protein family members detected in control samples, in particular HSP70 and HSP90 subfamily members. Negative control samples cannot reasonably detect a complete set of experimental contaminants, in large part due to inevitable variations in sample handling across the many steps prior to MS analysis. To account for this, candidate proteins were screened and filtered based on prevalence in negative control samples deposited within the CRAPome repository [40]. Consequentially, HSP family members that presented as candidate proteins were ultimately excluded, including the repeat identified (n=7) HSPA5 (HSP70 family) and (n=5) CCT8 (Chaperonin family) (Figure 7). Furthermore, multiple instances of HSP90 proteins (HSP90AB1, HSP90B1, HSP90AA1) were detected as candidate proteins prior to CRAPome filtering, consistent with reports describing HSP90 as a biochemically defined TIMP2 interacting partner [26]. The need to examine each level of the data empirically and thoroughly is further demonstrated through the likely detection of non-human proteins as proximal interactors, exemplified by F13A1. This matrisome-associated protein classically functions in the realms of blood coagulation, although it likely plays more diverse roles in the tissue microenvironment [73]. Assessment of the TIMP2 proximal interactome in human serum samples reveals a unique serum proximal interactome, through which F13A1 is identified amongst other unique proximal interactors. Based on the mechanistically undefined functions of TIMP2, and reports linking its activity with anti-tumor and anti-aging characteristics [6, 10–12, 74], it would be of great interest to assess whether the serum proximal interactomes of targets like TIMP2 could impart clinically relevant diagnostic information. Regardless, it is crucial that all the details concerning candidate identification (protein coverage, analysis scores, pre-defined protein function) be interrogated prior to further in-depth functional studies.

A common tool for analyzing PL data is SAINT (Significance Analysis of Interactome) [75]. We chose to forgo this method since SAINT uses only one metric (such as spectral count or peptide spectrum matches). SAINT analysis was not amenable to use of calculated protein abundance due to the large range in abundance values. Our analysis utilizes a simple series of calculations to assign order to the candidate interactors, considering both PSMs and abundance in samples and controls (see supplementary materials). To the ordered candidates, we assigned a threshold score (≥1) to determine candidate proteins to be filtered through CRAPome analysis. To prove the utility of our method, we also performed SAINT analysis on a duplicate experiment and compared the hits to our candidate proteins. This comparison revealed that our analysis was slightly more stringent in candidate protein selection, with SAINT analysis identifying two (TIMP2-TurboID) and nine (TurboID-TIMP2) additional candidate proteins when using Bayesian false discovery rate (BDFR) ≤0.05 (Table S8). SAINT analysis also allows users to incorporate known interactors into their analysis platform (topology-aware probability score), adding weight to protein IDs that are known interactors. We decided to exclude inference from previously identified interactors, and instead use our knowledge of known interactors to inform our determined threshold for candidate proximal interactors.

A key difference between the methods used to interrogate the proximal interactome of HS-5 versus HT1080 is the use of TMT labeling. In TMT experiments, the pipeline skews in favor of the control sample through rounds of normalization, starting with sample normalization at TMT labeling, then during normalization of total abundance in analysis. Theoretically, fusion protein samples should produce more biotinylated protein than the controls, something we corroborate with streptavidin blots. To account for skew in favor of control samples, and considering ratio compression that occurs in isobaric tagging methods [76], we utilize a low (1.5) fold change threshold for hit identification.

Here we present proof-of-principle for the utilization of extracellular proximity labeling (ePL) to investigate the proximal interactome of secreted factors. This method effectively identifies unique proximal interactor candidates in the matrisome compartment and can be adapted downstream for improved protein coverage. One such example may be to include a deglycosylation step prior to tryptic digestion that may enhance peptide discovery and quantification. Regardless, exploration of the interactome of matrisome components such as TIMP2 will provide important context with respect to their defined functions, and new research direction when exploring undiscovered functions. The protocol successfully identifies known interactors for TIMP2 (matrix metalloproteinases, HSP90 family members), and also reveals novel, highly reproducible interactors for further in-depth study. The matrisome represents an untapped resource with regards to therapeutic intervention in disease. To date, there are very few therapeutics that target the extensive, well-documented changes that occur in the extracellular matrix across the spectrum of human diseases. Continued investigation of the matrisome proximal interactome in model systems such as HT1080 and HS-5 cells will uncover interactome hubs within the extracellular compartment, potentially revealing novel therapeutic targets in human disease.

## Methods

### Construct design

All constructs were produced in a pBABE retroviral expression (Addgene plasmid# 80899, a gift from Kyle Roux) backbone using ligation-independent cloning (In-Fusion, Takara), and transformed into a recombination-deficient bacterial strain (NEB Stable competent cells, New England Biolabs). Insert containing clones were confirmed by restriction digest analysis and DNA sequencing. Large scale plasmid preps were performed with NucleoBond^®^ Xtra Maxi Plus purification kits (Macherey-Nagel). Detailed construct production can be found in the supplementary materials.

### Retroviral packaging and cell infection

HEK293T/17 cells (virus producing cells; VPCs) were grown to 80% confluence in 10cm tissue culture dishes. At the same time, target cells (HT1080, HS-5) were grown to 25% confluence. VPCs were transfected with 0.5ug pVSV-G (Addgene plasmid #8454, a gift from Bob Weinberg), 4.5ug pUMVC (Addgene plasmid #8449, a gift from Bob Weinberg), and 5ug of the desired pBABE expression vector. After 16–18 hours, the media on the VPCs was replaced with fresh DMEM 10% FBS, 1% GlutaMAX, 1% penicillin-streptomycin (FM) and 24 hours were allowed for virus production. The virus conditioned media of the VPCs was then collected and gently filtered through 0.45um polyethersulfone (PES) filters directly onto the target cells. The target cells were then supplemented with 4ug/mL polybrene. VPCs were supplemented with a fresh 10mL of FM and a second round of infection was performed the next day. Selection was initiated 8 hours after the final infection with 1ug/mL puromycin for 5 days. Fusion gene expressing cells were then expanded, characterized, and cryopreserved for future use.

### Generation of biotin-depleted full media (BDFM) and low-serum biotin-depleted media (LSBDM)

Biotin-depleted full media (BDFM) was generated by washing 2.5mL high-capacity streptavidin agarose (ThermoFisher #) four times with 50mL phosphate buffered saline, centrifuging the beads at 1000G for 2 minutes between washes. After the final wash, all of the beads were transferred to 1 liter of DMEM, 10% FBS, 1% GlutaMAX, 1% penicillin-streptomycin stored in a roller bottle. The media was then rolled at 4°C for 16–20 hours prior to filtration through a 0.22um PES filter unit. BDFM was used within 1 month of generation. Low-serum biotin-depleted media was generated by diluting the BDFM 1 in 10 with Advanced DMEM (Gibco).

### Extracellular proximity labeling assay

Fusion protein expressing or control cells were seeded in 15cm dishes (1.5×10^6^ HT1080, 7×10^6^ HS-5) in BDFM (media transfer cells; MTCs). At the same time, WT cells were seeded in 15cm plates in BDFM at the same density (receiver cells; RCs). If performing a spheroid-based ePL experiment, WT HT1080 were seeded at 10,000 cells per well of a 96-well ultra-low binding round bottomed spheroid microplate 3 days prior to seeding fusion protein-expressing or control cells (the latter grown in 2D). At 72 hours post seeding, MTCs were switched to 23mL LSBDM, while 10mL media on the RCs was replaced with fresh BDFM. **TurboID experiments**: On day 4, 24 hours after the previous step, the media from the RCs was discarded and replaced by the media on the MTCs. The remaining culture was supplemented with 1uM biotin, 0.1mM ATP and incubated for 1 hour. **BioID2 experiments**: On day 4, 24–30 hours after the previous step, the media from the RCs was discarded and replaced by the media on the MTCs. The remaining culture was supplemented with 1uM biotin and 2mM ATP and incubated for 16 hours. **Serum experiments:** Human blood was collected through the NIH Blood Bank, clotted for 1h at room temperature and centrifuged at 2000 × g for 10 minutes at 4°C and frozen. In a 1mL reaction, serum was diluted to 50% with Dulbecco’s phosphate buffered saline and supplemented with 300nM recombinant TurboID fusion protein, 1uM biotin, 0.1mM ATP, and incubated at 37°C for 1h. Reactions were stopped through desalting using PD-10 columns (Cytiva) and immediately incubated with streptavidin using the protocol described below.

### Lysate and media collection

Conditioned media was removed from the cells, supplemented with 0.1% protease inhibitor cocktail, and centrifuged at 1000 × G for 5 minutes to pellet cellular debris. The conditioned media was then concentrated to less than 1.5mL using Amicon Ultra-15 centrifuge tubes (MilliporeSigma), prior to buffer exchange of the concentrated media into Dulbecco’s phosphate buffered saline (DPBS) using PD-10 columns (Cytiva), resulting in 3.5mL of desalted media. Desalted media was concentrated again using Amicon Ultra-4 centrifuge tubes to a volume of 1mL. Cell lysate collection was performed at the same time as the above steps. Cells were placed on ice and washed 3x with ice-cold 12mL DBPS, followed by addition of 1250uL lysis buffer (50mM HEPES, 50mM NaCl, 2% SDS, 1% Triton X-100, 1% NP-40, 1% sodium deoxycholate, 10mM DTT, 1% protease inhibitor cocktail). The cells and matrix were scraped from the plate, then transferred to a 2mL centrifuge tube and mixed by vortexing (10 × 2 seconds). Lysate samples were then heated to 95°C for 10 minutes while shaking at 800 RPM and then cooled on ice for 3 minutes. Cooled samples were sonicated with 24× 5s bursts at 70% power, then centrifuged at 10,000 × G for 10 minutes. The supernatant was removed and combined with the desalted-concentrated conditioned media, before being snap-frozen in liquid nitrogen.

### Generation of trypsin-resistant streptavidin magnetic beads

Trypsin-resistant streptavidin magnetic beads were generated following the protocol described by Rafiee et. al. [77]. Briefly, 1.98mL of Sera-Mag Streptavidin Magnetic SpeedBeads (Cytiva) were washed with 4mL PBS 0.1% Tween-20 (PBS-T), then resuspended in 5.61mL 76.4mM cyclohexanedione in PBS-T, pH 13. The beads were then rotated for 4 hours at room temperature followed by a single wash in 4mL PBS-T. The supernatant was removed, then beads resuspended in 2.79mL 4% paraformaldehyde in PBS-T, then 2.79mL 0.2M Sodium cyanoborohydride in PBS-T was added. Note, these reagents are extremely toxic and handling should be performed in a chemical fume hood. The beads were rotated for 2 hours at room temperature, followed by supernatant removal, a single wash in 4mL 0.1M Tris-HCl (pH 7.5), then two washes with 4mL PBS-T. The beads were finally resuspended in 1.98mL PBS-T and stored at 4°C.

### Pulldown and sample preparation for mass spectrometry

Snap frozen sample were thawed in a 37°C water bath, then 60uL trypsin-resistant streptavidin magnetic beads were added to the whole sample. The samples were rotated at 4°C for 2 hours. After incubation, beads were allowed to bind to the magnet for 5 minutes, followed by supernatant (output) removal and two washes with 1mL wash 1 (2% SDS). This was followed by two individual 1mL washes with wash 2 (0.1% sodium deoxycholate, 1% Triton X-100, 500mM NaCl, 1mM EDTA, 50mM HEPES) and wash 3 (250mM LiCl, 0.5% NP-40, 0.5% sodium deoxycholate, 1mM EDTA, 10mM Tris pH 8), followed by two washes with 1mL wash 4 (20mM HEPES, 150mM NaCl). After supernatant removal, a single wash with 500uL 100mM triethylammonium bicarbonate (TEAB) was performed and sample transferred to a fresh lo-bind 1.5mL tube (Eppendorf), at which point the beads were re-suspended in 50uL 100mM TEAB, 5mM DTT and incubated at 56°C for 25 minutes to reduce the samples. Samples were then supplemented with 1.55uL 0.5M iodoacetamide (15mM working concentration) and incubated at room temperature in the dark for 30 minutes. The supernatant was removed, and samples washed three times with 200uL 100mM TEAB, then finally resuspended in 30uL 100mM TEAB + 0.125ug Trypsin-LysC mixture (Promega) and incubated for 16–18 hours at 37°C with shaking at 500 RPM. Samples were centrifuged at 500 × G for 20 seconds, then placed on the magnet and the supernatant was transferred to a fresh 1.5mL Lo-bind tube. **For unlabeled processing**: the remaining beads were washed with 30uL 100mM TEAB, then supernatants combined. Samples were acidified with 3uL 10% trifluoroacetic acid (TFA) and snap frozen in liquid nitrogen. **For TMT-labeling**: The remaining beads were washed with 15uL 100mM TEAB, then supernatants combined. Peptide concentration was determined using A205, then sample concentrations normalized. Samples were then supplemented with 3.64ug/uL TMT reagents and incubated in the dark for 1 hour at room temperature. Each sample was supplemented with 1uL 15% hydroxylamine, then 1.7uL 10% trifluoroacetic acid. Samples were then combined into a single tube. The individual sample was then cleaned and concentrated using 100uL C18 pipette tips (ThermoFisher) and eluted in 0.1% formic acid, 95% acetonitrile prior to snap-freezing in liquid nitrogen.

### Mass spectrometry

Before analysis, samples were dried using a SpeedVac system (Thermo Scientific) and resuspended in 16uL 0.1% formic acid, with 12uL being analyzed by liquid chromatography and tandem mass spectrometry (LC-MS/MS). The LC-MS/MS analysis of tryptic peptides for each sample was performed sequentially with a blank run between each two sample runs using a Thermo Scientific Orbitrap Exploris 240 Mass Spectrometer and a Thermo Dionex UltiMate 3000 RSLCnano System. Peptides from trypsin digestion were loaded onto a peptide trap cartridge at a flow rate of 5μL/min. The trapped peptides were eluted onto a reversed-phase Easy-Spray Column PepMap RSLC, C18, 2μM, 100A, 75μm × 250 mm (Thermo Scientific) using a linear gradient of acetonitrile (3–36%) in 0.1% formic acid. The elution duration was 110 min at a flow rate of 0.3μL/min. Eluted peptides from the Easy-Spray column were ionized and sprayed into the mass spectrometer, using a Nano Easy- Spray Ion Source (Thermo Scientific) under the following settings: spray voltage, 1.6 kV, Capillary temperature, 275°C. Other settings were empirically determined. **For unlabeled samples**: Raw data files were searched against human protein sequences using the Proteome Discoverer 3.0 software (Thermo Scientific) based on the SEQUEST algorithm. Carbamidomethylation (+57.021 Da; cysteine) was set as static modifications. Oxidation/hydroxylation +15.995Da (methionine, proline, lysine), and deamidation +0.984 Da (asparagine, glutamine) were set as dynamic modifications. The minimum peptide length was specified to be five amino acids. The precursor mass tolerance was set to 15 ppm, whereas fragment mass tolerance was set to 0.05 Da. The maximum false peptide discovery rate was specified as 0.01. **For TMT-labeled samples**: The Exploris 240 instrument was operated in the data dependent mode to automatically switch between full scan MS and MS/MS acquisition. Survey full scan MS spectra (m/z 350−1800) was acquired in the Orbitrap with 35,000 resolutions (m/z 200) after an accumulation of ions to a 3 × 10^6^ target value based on predictive automatic gain control (AGC). The maximum injection time was set to 100 ms. The 20 most intense multiply charged ions (z ≥ 2) were sequentially isolated and fragmented in the octopole collision cell by higher-energy collisional dissociation (HCD) using normalized HCD collision energy 30 with an AGC target 1×10^5^ and a maxima injection time of 400 ms at 17,500 resolutions. The isolation window was set to 2 and fixed first mass was 120 m/z. The dynamic exclusion was set to 30 s. Charge state screening was enabled to reject unassigned and 1+, 7+, and >7+ ions. Raw data files were searched against human protein sequences using the Proteome Discoverer 3.0 software (Thermo Scientific) based on the SEQUEST and percolator algorithms. Carbamidomethylation (+57.021 Da; cysteine) and TMT labeling (+229.163 Da; N-terminus, lysine) was set as static modifications.

Oxidation/hydroxylation +15.995Da (methionine, proline, lysine), and deamidation +0.984 Da (asparagine, glutamine) were set as dynamic modifications. The minimum peptide length was specified to be five amino acids. The precursor mass tolerance was set to 15 ppm, whereas fragment mass tolerance was set to 0.05 Da. The maximum false peptide discovery rate was specified as 0.01. The resulting Proteome Discoverer Report contains all assembled proteins with peptides sequences, peptide spectrum match counts (PSM), and TMT-tag based quantification using reporter ion abundance.

### Proximal interactor candidate selection

*For unlabeled experiments*: We performed candidate protein selection using a series of basic calculations comparing peptide spectrum matches (PSMs) and calculated protein abundance between fusion gene and control samples (summarized in supplementary files materials, calculations maintained in supplementary tables). *For TMT-labeled experiments*: total abundance of each sample is normalized (1 control, 2 fusion protein samples), prior to fold change comparison between fusion protein samples versus the control sample. Proteins with an abundance ≥ 1.5 times the control are identified as candidate proteins.

Final protein candidates are identified for filtering against the Contaminant Repository for Affinity Purification (CRAPome), with any protein candidate that is detected in over 99 (out of 716) control experiments within the CRAPome database automatically excluded.

### Significance of Interactome (SAINT) Analysis

Bait, prey, and interaction files from a duplicate ePL experiment (2D HT1080 TurboID experiment, unstimulated) were uploaded in to the SAINTexpress tool on the APOSTL Galaxy Server (apostl.moffitt.org) [78]. SAINTexpress significant hits were identified as proteins with a Bayesian false discovery rate (BFDR) of ≤ 0.05.

### Co-localization

2×10^5^ HT1080 cells were seeded in to 3-well removable chamber slides (Ibidi) and cultured overnight. Next day, the cells were treated with 0.1ug/mL recombinant TIMP2 (to mimic the over-expression of fusion proteins) for 1h. Media was removed from the cells prior to fixation in 4% PFA without washing. Fixed cells were permeabilized with PBS 0.1% Triton X-100, then blocked in PBS 5% donkey serum for 30 minutes. Cells were then stained with primary antibodies for 2h, washed 3x with PBS, then stained with secondary antibodies for 1h and stained with 600nM DAPI for 2 minutes prior to mounting. Antibody details are supplied in supplementary materials. 63X images were collected using a Zeiss LSM880 confocal microscope, 40X images were collected using a Zeiss Axio Observer 5 multimodal imaging microscope.

### Cloning of recombinant TurboID and TIMP2 fusion proteins

Vectors were created by Gateway multisite recombination reactions using the methods described in Wall *et. al*. [79] and pDest-303 (Addgene #159678). The optimized CMV promoter Entry clone used can be obtained from Addgene (#162927) and the three TIMP2 signal sequence driven Entry clones for TurboID-His6, TIMP2–13xG4S-TurboID-His6, and TurboID-His6-13xG4S-TIMP2 (31351-E03) were synthesized by ATUM [gene-optimized for human embryonic kidney (HEK) cell expression]. Maxiprep DNA for all three constructs was generated using the Plasmid Plus Maxi Kit (Qiagen).

### Expression and purification of recombinant TurboID and TIMP2 fusion proteins

All protein samples were processed following the same protocol. One liter of Expi293F (Gibco) culture supernatant was harvesting using established protocols. Expression time was 96 hours. Supernatant was stored at −80°C prior to protein purification. Culture supernatant was concentrated using a Amicon stirred cell concentrator (EMD Milipore) with a 10 kDa molecular weight cut off membrane. The volume of the supernatant was reduced by half and dialyzed against 20 mM HEPES, ph 7.4, 300 mM NaCl and filtered through an Acropack 500 capsule 0.8/0.2 μm filter. All purification steps were performed on a Bio-Rad NGC chromatography system at room temperature. A 10-ml HisTrap FF column (Cytiva) was equilibrated in 10 column volumes (CV) 95% Buffer A (20 mM HEPES, pH 7.4, 300 mM NaCl) and 5% Buffer B (Buffer A with 500 mM imidazole). Filtered culture supernatant was loaded onto the column followed by 10 CV wash in 5% Buffer B. Protein was eluted from the column with bump to 100% Buffer B.

Fractions were collected across the gradient and analyzed by SDS-PAGE/Coomassie Blue staining. Elution fractions containing target protein were pooled.

## Supplementary Material

1 Figure S1. Gelatin zymography of conditioned media from HT1080 cells treated with concanavalin A or PMA for 24h.
**Figure S2. Supplemental immunofluorescence images.** (A) Control images from cells immunostained in identical conditions to dual-stained cells, but with one primary antibody. Images reveal no bleeding between fluorophores or background staining from the secondary antibodies. (B) Supplemental 40X images showing co-localization between TIMP2 and CCN1/CCN2/THBS1, showing that co-localization is not uniform across all cells.

## Footnotes

Supplementary Files This is a list of supplementary les associated with this preprint. Click to download.

- Supplementarymaterials.docx
- TableS1.xlsx
- TableS2.xlsx
- TableS3.xlsx
- TableS4.xlsx
- TableS5.xlsx
- TableS6.xlsx
- TableS7.xlsx
- TableS8.xlsx

## Abbreviations

ADAM: A disintegrin and metalloproteinases
ADAMTS: ADAM with thrombospondin type 1 motifs
CCN1/2: cellular communication network factor 1/2
ConA: Concanavalin A
CRAPome: contaminant repository for affinity purification–mass spectrometry
ECM: Extracellular matrix
ePL: Extracellular proximity labeling
MMP: Matrix metalloproteinase
MP: Metalloproteinase
PL: Proximity labeling
PMA: Phorbol 12-myristate 13-acetate
THBS1: Thrombospondin 1
TIMP: Tissue inhibitor of metalloproteinase
