# Supplementary material for: Extracellular Proximity Labeling Reveals an Expanded Interactome for the Matrisome Protein TIMP2
Source: Res Sq. 2024 Jan 15:rs.3.rs-3857263. Preprint. [Version 1] doi: 10.21203/rs.3.rs-3857263/v1 (PMC10836090; doi:10.21203/rs.3.rs-3857263/v1)
Supplement: 1 — Figure S1. Gelatin zymography of conditioned media from HT1080 cells treated with concanavalin A or PMA for 24h. Figure S2. Supplemental immunofluorescence images. (A) Control images from cells immunostained in identical conditions to dual-stained cells, but with one primary antibody. Images reveal no bleeding between fluorophores or background staining from the secondary antibodies. (B) Supplemental 40X images showing co-localization between TIMP2 and CCN1/CCN2/THBS1, showing that co-localization is not uniform across all cells. [file NIHPPRS3857263V1-supplement-1.pdf]

HT1080 Conditioned media (24 hours post treatment)

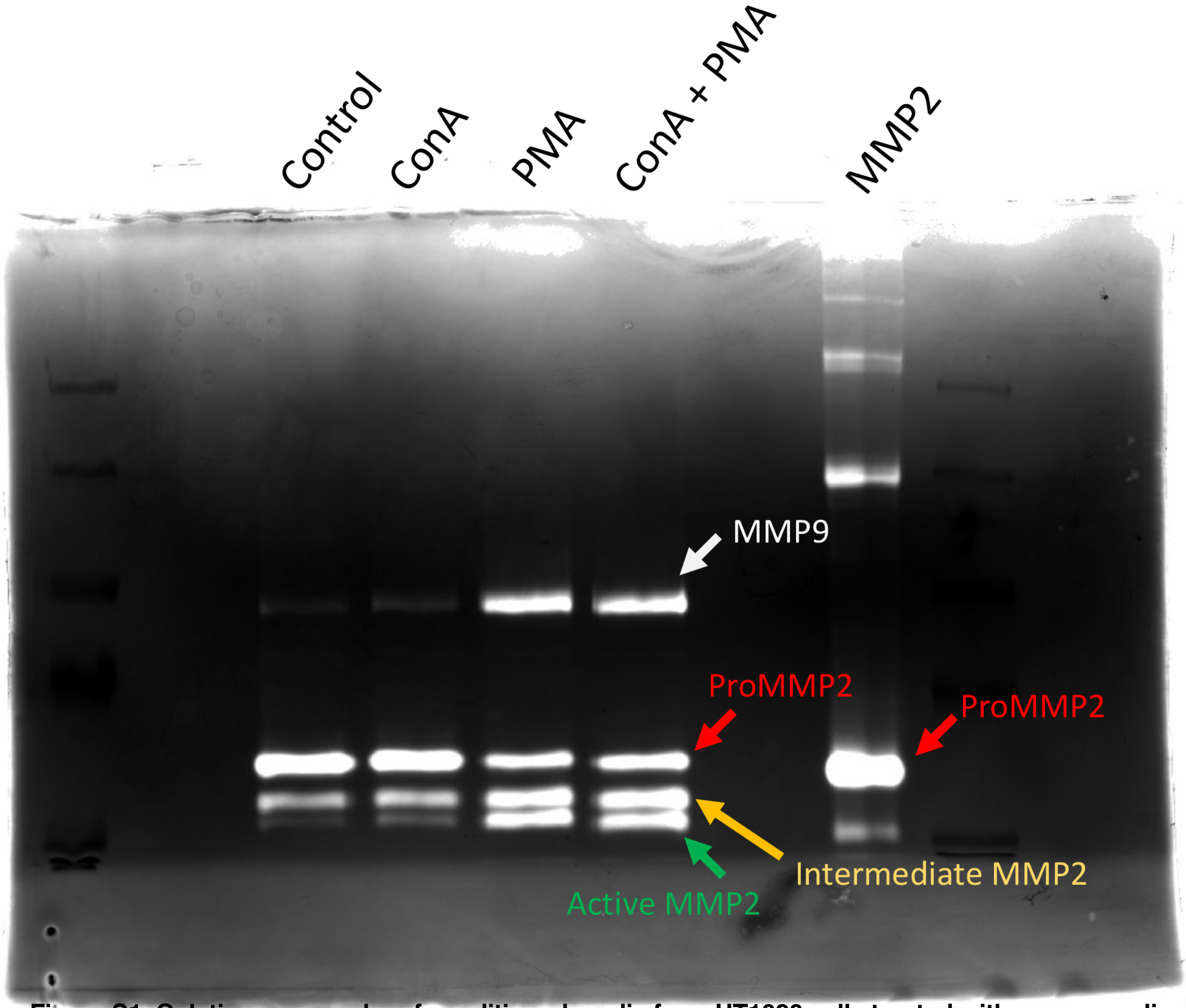

Figure S1. Gelatin zymography of conditioned media from HT1080 cells treated with concanavalin A or PMA for 24h.

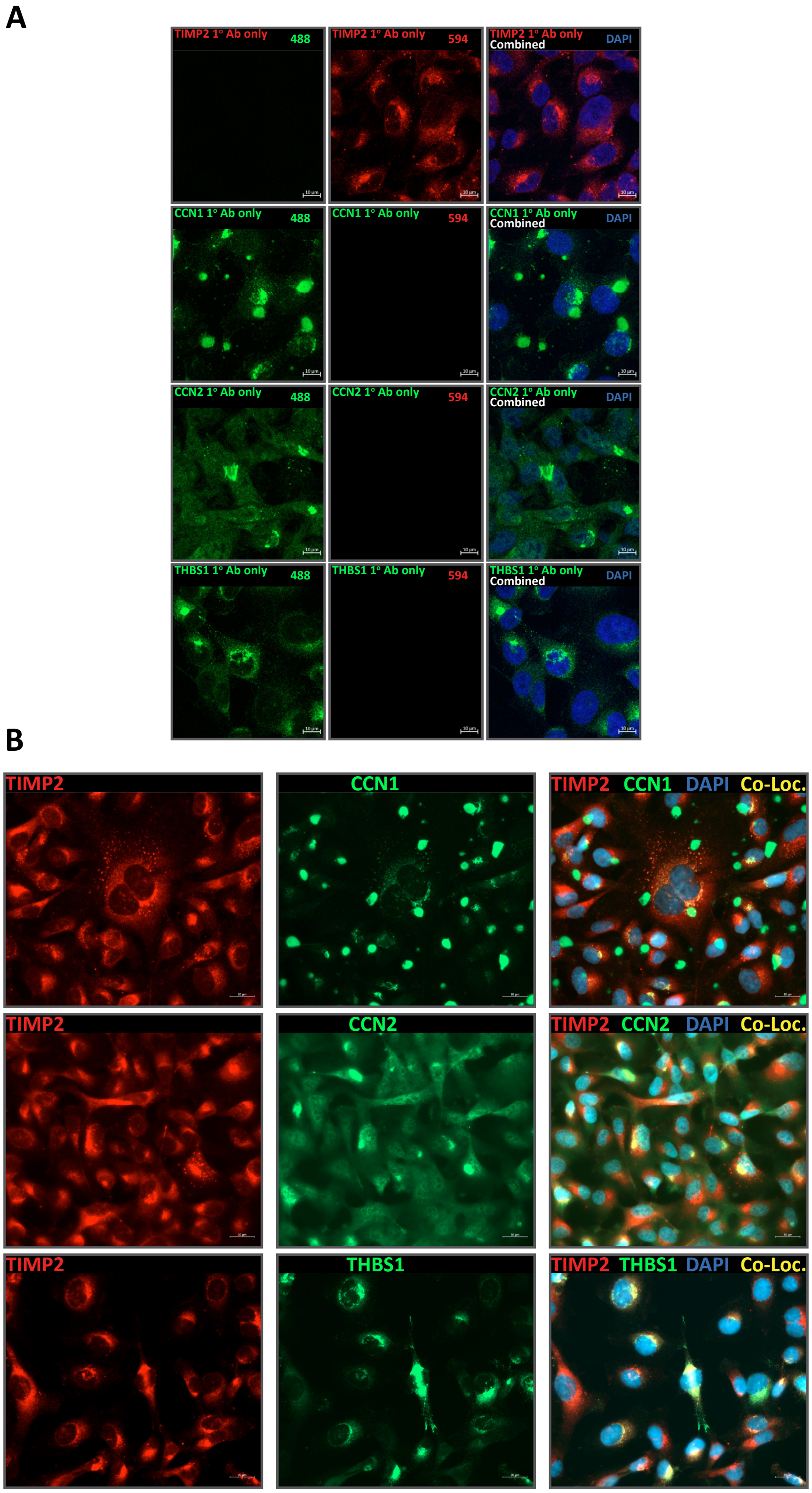

Figure S2. Supplemental immunofluorescence images. (A) Control images from cells immunostained in identical conditions to dual-stained cells, but with one primary antibody. Images reveal no bleeding between fluorophores or background staining from the secondary antibodies. (B) Supplemental 40X images showing co-localization between TIMP2 and CCN1/CCN2/THBS1, showing that co-localization is not uniform across all cells.
